# Arabic Adaptation and Validation of the SARC-F Questionnaire for Sarcopenia Screening in Elderly Populations: Exploration of Associated Factors

**DOI:** 10.1155/jare/7405872

**Published:** 2025-08-08

**Authors:** Hadeel M. Badwan, Ziad Hawamdeh, Mohamed I. Mabrouk, Mohamed I. Kamel, Alia A. Alghwiri

**Affiliations:** ^1^Department of Physical Therapy, Faculty of Applied Medical Sciences, Al-Zaytoonah University of Jordan, Amman, Jordan; ^2^School of Medicine, University of Jordan, Amman, Jordan; ^3^Department of Physiotherapy, Faculty of Allied Medical Sciences, Applied Science Private University, Amman, Jordan; ^4^University of St. Augustine for Health Sciences, Augustine, Florida, USA; ^5^Department of Paediatrics and its Surgery, Faculty of Physical Therapy, Cairo University, Giza, Egypt; ^6^Department of Physiotherapy, School of Rehabilitation Sciences, University of Jordan, Amman, Jordan

## Abstract

**Background:** Sarcopenia is a geriatric condition marked by decreased muscle mass and function as people age. The SARC-F questionnaire is a simple and useful instrument for sarcopenia screening but it is not available in the Arabic language. This study aimed to translate the SARC-F into the Arabic language, validate it among Arabic-speaking older adults, and explore the association between SARC-F and fatigue, QOL, and cognitive impairment.

**Methods:** SARC-F was translated into Arabic according to WHO guidelines, and older adults aged 60 years and older were recruited. Test–retest reliability of SARC-F was examined over a 2-week period. SARC-F was validated based on the revised European Working Group on Sarcopenia in Older People criteria. Sensitivity, specificity, and positive and negative predictive values were assessed against bioelectrical impedance analysis (BIA). The association between the Arabic SARC-F and Modified Fatigue Impact Scale, Medical Outcomes Study Short Form 12, and Montreal Cognitive Assessment was investigated too.

**Results:** Eighty-six older adults participated in this study (59.3% females). The Arabic SARC-F showed an intraclass correlation coefficient (ICC) of 0.926 (95% CI = 0.88–0.95) and Cronbach's alpha of 0.81. Sensitivity, specificity, and positive predictive value and negative predictive value were 36.4%, 78.7%, 20%, and 89.3%, respectively. The Arabic version of SARC-F showed good reliability and validity.

**Conclusion:** The Arabic SARC-F is a valid and reliable tool for sarcopenia screening, showing a good ability to identify individuals with sarcopenia and predict the absence of the condition. The Arabic SARC-F was associated with fatigue and QOL but not with cognitive impairment. These results support the use of the Arabic SARC-F as a useful questionnaire for sarcopenia screening in Arabic-speaking populations.

## 1. Introduction

Sarcopenia is a common disorder in older adults characterized by a loss of skeletal muscle mass, reduced muscle power, as well as poor physical performance [1]. This condition leads to several adverse outcomes, including the deterioration of the quality of life (QOL) [2], reduced the ability to perform activities of daily living [3], and an increased risk of falls [4]. Furthermore, sarcopenia is associated with fatigue [5], mental impairments [6], and increased mortality [7]. Several definitions and diagnostic criteria for sarcopenia have been proposed [8]. However, all criteria require the assessment of muscle mass, muscle strength, and/or physical performance [1]. Therefore, the diagnostic procedures for sarcopenia should include measuring muscle mass, muscle strength, and functional ability [1].

Several tools have been used to measure muscle mass of the body such as the magnetic resonance imaging (MRI), computed tomography scan (CT-scan), dual energy X-ray absorptiometry (DXA), or bioelectrical impedance analysis (BIA) [9], whereas muscle strength can be measured using the handgrip dynamometer and chair stands test [10]. The Short Physical Performance Battery (SPPB) and gait speed are commonly used to determine physical capacity [11]. The use of MRI, CT-scan, or DXA to determine muscle mass for the initial detection of sarcopenia in any patient with suspected symptoms is expensive and time-consuming, and needs technical training. This limits the feasibility of using the suggested diagnostic procedures as a routine investigation in older adults. Furthermore, those methods were designed to diagnose sarcopenia but not to screen for sarcopenia. Therefore, the need for a simple, rapid, and inexpensive sarcopenia screening tool has risen.

Malmstrom and Morley developed the SARC-F questionnaire, as a fast, low-cost, and simple sarcopenia screening tool [12]. The SARC-F is a practical instrument for sarcopenia screening in everyday clinical practice [12]. Furthermore, the European Working Group on Sarcopenia in Older People (EWGSOP) recommended using the SARC-F questionnaire as the initial step in the sarcopenia diagnosis process in their revised consensus (EWGSOP2) [13]. Muscle power, walking assistance, rising from a chair, ascending stairs, and falling are the five items included in the SARC-F questionnaire [12]. The cut-off score in the total score of the SARC-F is “4.” The SARC-F has been translated and validated into several languages to be used in screening cases of probable sarcopenia in different countries. However, it is not available in Arabic, limiting its utility in Arabic-speaking regions.

Given that Arabic is the first language for over 400 million people across 22 countries, there is a clear need for validating sarcopenia screening tools in Arabic. Additionally, the association between the SARC-F score and outcomes such as fatigue, QOL, and cognitive impairments has been underexplored in these populations. The absence of an Arabic version of the SARC-F presents a significant gap. Arabic-speaking healthcare providers face difficulties in diagnosing and managing sarcopenia due to the lack of culturally and linguistically appropriate screening tools. This gap can impede effective patient assessment and management, especially in regions where diagnostic resources are limited. For instance, healthcare providers in Arabic-speaking countries often struggle with limited access to validated tools, leading to delays in diagnosis and treatment. Furthermore, patients may face barriers in understanding and completing screening tools, which can impact early detection and intervention.

This study aims to translate and validate the SARC-F into Arabic and explore its association with fatigue, QOL, and cognitive impairment in Arabic-speaking older adults. An Arabic version of the SARC-F would enhance sarcopenia screening in clinical practice, raise awareness among healthcare providers, and reduce reliance on costly and invasive diagnostic procedures.

## 2. Methods

### 2.1. Study Population and Design

A cross-sectional study was conducted to investigate the psychometric properties of the Arabic SARC-F. This study had the ethical approval from Jordan University Hospital Institutional Review Board (IRB reference number 150/2021). A consent form that explains all study procedures was signed from all participants before the conduction of the study.

A convenience sample of 86 older adults was recruited in this study. The inclusion criteria included any individual who is at the age of 60 years or older from both sexes. Exclusion criteria included people who were unable to read and write in Arabic and who have serious physical and/or mental disabilities that prevented them from pursuing the study procedures. Eligible older adults were invited for the examination that took around 1 h at the School of Rehabilitation Sciences, University of Jordan. Data were collected from May to December 2022.

Although the sample size may appear small, it was sufficient for conducting preliminary analyses and obtaining meaningful insights into the translation and cultural adaptation process. A power analysis was performed to evaluate the adequacy of the sample size. The analysis aimed to detect moderate effect sizes (*r* ≈ 0.3) with a power of 0.80 and an alpha level of 0.05. The power analysis suggested that a sample size of approximately 85 participants would be sufficient to detect significant correlations and differences. Thus, the sample size of 86 participants was considered appropriate for the study's exploratory nature.

### 2.2. Translation/Validation Processes

Permission to translate the SARC-F into the Arabic language was granted from the authors before starting the study. The English version of the SARC-F was translated into the Arabic language in accordance with the World Health Organization (WHO) forward/backward translation protocol and international standards [14]. Two stages have been conducted: (1) the translation and cross-cultural adaptation of the SARC-F in the Arabic language and (2) the clinical validation of the Arabic SARC-F with other outcome measures.

#### 2.2.1. Translation and Cultural Adaption

The translation stage was accomplished through several phases [14]. In the first phase, the SARC-F questionnaire, originally in English, was translated into Arabic by a bilingual therapist fluent in both languages. This initial translation focused on maintaining the semantic meaning of the original content, ensuring that the questions were linguistically accurate and comprehensible to Arabic-speaking populations. The therapist was also mindful of cultural nuances that might affect the interpretation of specific terms or concepts within the questionnaire. In the second phase, a panel of experts that included physiotherapists and physicians reviewed the translated version and checked the conceptual appropriateness and consistency of the translation and prepared an edited Arabic version. During this stage, the experts identified any terms or phrases that could be culturally misinterpreted or were not commonly used in the Arabic-speaking context. For instance, units of measurement were converted from pounds to kilograms to align with the metric system used in Arab countries. The panel then refined and edited the translation to produce an improved Arabic version of the SARC-F. In the third phase, an English bilingual expert translator (who is unaware of the initial English version of the questionnaire) translated the Arabic SARC-F into the English language. The back-translated version was then compared to the original English version to assess the fidelity of the translation. In the fourth phase, the same panel of experts met again to compare the original English version of SARC-F to the back-translated version and prepared an edited Arabic version. Afterward, the edited Arabic version of SARC-F was applied to 10 older adults (5 males and 5 females) to pilot test the clarity of the Arabic version and define any cultural adaptations that need to be made. The pilot testing confirmed that the Arabic version was clear and culturally appropriate and effectively captured the intended content of the original SARC-F. Based on this feedback, the final Arabic version of the SARC-F was developed and deemed ready for use.

#### 2.2.2. Reliability

##### 2.2.2.1. Test–Retest Reliability

Test–retest reliability was performed in order to measure the consistency of results over time without a real change in individual's status. The questionnaire was self-administered and took approximately 2 min to complete. To examine the test–retest reliability of the Arabic SARC-F, it was administered twice within a 2-week interval [15]. The 2-week period was selected since it is long enough to eliminate the recall bias while still being short enough to avoid major changes in the participants' physical capability. The intraclass correlation coefficient (ICC 3.1) with a 95% confidence interval (CI) was used to evaluate reliability.

##### 2.2.2.2. Internal Consistency

In order to evaluate the degree of homogeneity among the items of the Arabic SARC-F internal consistency was estimated using Cronbach's alpha.

#### 2.2.3. Validation Process

Following the translation of the SARC-F into the Arabic language, a clinical validation assessment was conducted for the Arabic SARC-F to determine its performance in terms of sarcopenia screening according to EWGSOP2 definition.

##### 2.2.3.1. Concurrent Validity

Despite that using DXA is more precise for sarcopenia diagnosis, BIA is considered as an acceptable technique for the diagnosis of sarcopenia as well [13]. DXA and BIA had high concurrent validity (*r* = 0.79) and high reliability (ICC > 0.95) [16]. In the current study, we used BIA as the gold standard for sarcopenia diagnosis because it is inexpensive and portable and does not expose participants to radiation. Concurrent validity was assessed using the association between the Arabic SARC-F and BIA results.

##### 2.2.3.2. Convergent Validity

The correlation between the Arabic SARC-F and grip strength, SPPB, gait speed, and chair rises (5-times sit-to-stand) were conducted to assess convergent validity.

### 2.3. The Association Between SARC-F and Other Health-Related Measures

The association between the Arabic SARC-F and Modified Fatigue Impact Scale (MFIS), Medical Outcomes Study Short Form 12 (SF-12), and Montreal Cognitive Assessment (MOCA) was conducted. Permission has been granted to use MOCA in this study.

### 2.4. Measurements and Instruments

The following information were collected from all participants including age, gender, height, weight, educational level, smoking, other diseases, and medications. Additionally, they were asked relevant questions about falling in the previous year (have you fallen in the past year? If yes, how many times has this happened?). This data collection helped contextualize the findings and assess potential confounding factors.

The BIA test was conducted for the study participants to determine participants with sarcopenia (skeletal muscle mass index < 5.5 for women and < 7.0 for men). BIA is an effective and acceptable approach for the diagnosis of sarcopenia based on the European working group [15]. Sarcopenia diagnosis has been estimated using several equations that were suggested by the EWGSOP2. The most commonly used equation utilizes the appendicular skeletal muscle mass (ASM) and individual's height (ht) [ASM/ht^2^] [17]. A cut-off score for a confirmed diagnosis of sarcopenia in women was suggested as (ASM/ht^2^) < 5.5 and (ASM/ht^2^) < 7.0 for men according to the updated EWGSOP2 criteria [15]. It was found that BIA has high concurrent validity with DXA (*r* = 0.79) and high reliability (ICC > 0.95) [16]. For the BIA, we used InBody 270 device for muscle mass analysis. Then, they completed the Arabic SARC-F and the recommended outcome measures by the EWGSOP2 including grip strength, SPPB, gait speed, and chair rises.

Grip strength assessment is an important part in the process of sarcopenia screening [18]. The final grip strength score can be estimated by taking the average of 3 trials with rest in between which was reported as the most reliable way [19]. The SPPB was found to be useful in identifying older adults with severe sarcopenia [20]. Gait speed is extensively utilized in practice because it is rapid, safe, and highly reliable diagnostic measure for sarcopenia [18]. A measure of gait speed over a distance of 4 m at the participant's usual walking speed was utilized in this study. The cut-off scores for screening of sarcopenia were determined for men as grip strength of less than 27.0 kg and for women as less than 16.0 kg, SPPB ≤ 8 points indicate severe sarcopenia, a cut-off score of gait speed ≤ 0.8 m/s in both genders indicates severe sarcopenia, and a cut-off score of chair-rise test was set as more than 15 s to have possible sarcopenia according to the updated EWGSOP2 criteria [13]. Finally, they completed other health outcome measures including MFIS, SF-12, and MOCA for further validation of the Arabic SARC-F.

### 2.5. Statistical Analysis

Descriptive statistics was implemented to calculate demographical and health-related information including mean and standard deviation (SD) or median and interquartile range (IQR) for continuous data based on its normality, and frequency and percentage for categorical variables. The Shapiro–Wilk test was used to evaluate the normality of the variable distribution.

The test–retest reliability of the SARC-F was calculated using the intraclass correlation coefficient (ICC 3.1) and 95% CI [18]. Cronbach's alpha was used to test the internal consistency of the Arabic SARC-F. Spearman correlation coefficient was used to calculate the association between study variables because the data were not normally distributed. Spearman correlations were interpreted using the following criteria: perfect correlation (+1/−1), strong correlation (0.9–0.7), moderate correlation (0.6–0.4), and weak correlation (0.3–0.1) [21]. SPSS Version 21 was used for the analysis.

The validity of a screening test is usually described using sensitivity, specificity, positive predictive value (PPV), and negative predictive value (NPV) [22]. In this study, the previously mentioned values were estimated for the Arabic SARC-F compared to the results of the gold standard for confirming the diagnosis of sarcopenia which is the BIA in accordance with the EWGSOP2 criteria calculations.

## 3. Results

### 3.1. Participants' Characteristics

A convenience sample of 86 older adults aged 60 years and older was recruited in this study, with a median age of 65 (IQR = 8) and 59.3% females. Twenty participants (23.3%) had a positive SARC-F score of ≥ 4 points, whereas 11 participants (12.8%) were identified as sarcopenic older adults based on the BIA index according to EWGSOP2. The descriptive statistics of the sample demographics and health related variables are presented in Table 1.

### 3.2. Translation and Cultural Adaptation

An Arabic version of the SARC-F was developed using the WHO guidelines. Cultural adaptation involved modifying specific terms and examples to align with cultural norms and practices. For example, in the original SARC-F, the weight measurement was stated in “pounds” (lbs), which is not commonly used in Jordan and other Arab countries. Instead, the measurement was converted to “kilograms” (kg), as this is the standard unit of weight in the region. Therefore, the question was adapted to ask, “How much difficulty do you have in lifting and carrying 5 kg?” The adapted Arabic version of the SARC-F was then subjected to pilot testing with a small sample of the target population. Participants were asked to complete the questionnaire and provide feedback on its clarity, relevance, and any difficulties they experienced in understanding the questions. After incorporating the feedback and making necessary adjustments, the Arabic version of the SARC-F was finalized.

### 3.3. Reliability

The test–retest reliability of the SARC-F was assessed in 78 participants. There were eight participants who did not complete the SARC-F for the second time. The results of test–retest reliability using the ICC and 95% CI for items and total score of the Arabic SARC-F are presented in Table 2. Results show that the Arabic version of the SARC-F has excellent test–retest reliability, with an ICC for the total score of 0.926 (95% CI: 0.884–0.953). This high level of agreement suggests that the instrument is stable over time. The internal consistency of the Arabic SARC-F using Cronbach's alpha was 0.81, which indicates a good internal consistency of the Arabic SARC-F [23].

### 3.4. Validity

According to EWGSOP2 criteria, 20 participants (23.3%) had a positive SARC-F score of ≥ 4 points, whereas 11 participants (12.8%) were diagnosed with sarcopenia based on the BIA index (ASM/ht^2^) cut-off scores. Distribution of samples using the Arabic version of the SARC-F questionnaire and the EWGSOP2 criteria are presented in Table 3. The results of sensitivity and PPV were 36.4% and 20%, respectively. This means that the Arabic SARC-F has low ability to correctly exclude people who do not have sarcopenia (high false negative); that is, individuals who test negative on the Arabic SARC-F (less than 4) might have sarcopenia. Additionally, the probability of having a positive test on the Arabic SARC-F in people who really have sarcopenia is only 20%, whereas the results of specificity and NPV were 78.7% and 89.3%, respectively. This means that the Arabic SARC-F has high ability to correctly detect people who have sarcopenia (few false positive); that is, individuals who test positive on the Arabic SARC-F (4 and above) are likely to have sarcopenia. Additionally, the probability of having a negative test on the Arabic SARC-F in people who do not have sarcopenia is 89.3%.

The low sensitivity and PPV suggest that while the Arabic SARC-F is useful for ruling out sarcopenia, it may not be sufficient as a standalone diagnostic tool. Clinicians should consider using it in conjunction with other diagnostic methods, such as physical performance tests or imaging, to improve diagnostic accuracy.

The convergent validity between the Arabic SARC-F total score and the main variables that were described by the guidelines of the EWGSOP2 (SPPB, gait speed, grip strength, and chair stands) to detect sarcopenia was established in this study (Table 4). We found inverse and moderate correlation between SARC-F total score and SPPB (*r* = −0.410, *p* < 0.001), weak inverse correlation between SARC-F total score and gait speed (*r* = −0.396, *p* < 0.05), weak inverse correlation between SARC-F total score and grip strength in the right and left sides (*r* = −0.271, *r* = −0.244, *p* < 0.05, respectively), and significant weak correlation between SARC-F total score and chair stands (*r* = 0.351, *p* < 0.001) as shown in Table 4.

### 3.5. The Association Between SARC-F and Other Health-Related Measures

The association between the SARC-F total score and the MFIS, the individual domains of SF-12, and MOCA are illustrated in Table 5. The highest correlation was observed between the SARC-F total score and SF-12 physical subscale (*r* = −0.679, *p* < 0.001). The SARC-F total score was significantly and moderately correlated with the MFIS total score (*r* = 0.537, *p* < 0.001). No significant correlation was found between the SARC-F total score and MOCA (*r* = −0.190, *p* < 0.05). Significant but weak correlation was found between the SARC-F total score and both ASM (*r* = −0.272, *p* < 0.05) and BMI (0.270, *p* < 0.05). However, no significant correlation was found between the SARC-F total score and ASM/height^2^ (*r* = −0.179, *p* > 0.05).

## 4. Discussion

### 4.1. Reliability

The Arabic version of the SARC-F showed an excellent test–retest reliability and good internal consistency, with an ICC of 0.926 (95% CI = 0.88–0.95) and Cronbach's alpha of 0.81. The Arabic SARC-F reliability results are a little higher than those observed from evaluating the initial English version of the SARC-F in three cohort studies by Malmstrom et al. [3] where the ICC was 0.79 in the Nutrition Examination Survey, 0.78 in Baltimore Longitudinal Study of Aging, and 0.81 in the African American Health study. In the study by Drey and colleagues, the German version of the SARC-F showed good test–retest reliability with an ICC of 0.899 (0.95% CI 0.784–0.954) and acceptable Cronbach's alpha of 0.67 [15], but it was a little lower than our findings. However, Krzymińska-Siemaszko et al. reported that the test–retest reliability of the Polish version of SARC-F was excellent with an ICC of 0.928, which is similar to our finding, but they reported lower Cronbach's alpha (0.784) than ours [24]. The high ICC of 0.92 for the total score of Arabic SARC-F that we had in our study indicates that the Arabic SARC-F provides consistent results overtime. Additionally, a Cronbach's alpha of 0.81 for the Arabic SARC-F reflects that the items in the test are highly correlated and homogenized.

The development and validation of the Arabic SARC-F have significant clinical implications for healthcare professionals working with Arabic-speaking populations. The high test–retest reliability and good internal consistency of the Arabic SARC-F suggest that it is a stable and coherent tool for assessing sarcopenia. Clinicians can confidently use the Arabic SARC-F in repeated assessments, knowing that the results are likely to be reliable.

### 4.2. Validity

In this validation study, the Arabic SARC-F had low sensitivity (36.4%) using EWGSOP2 criteria. This means that it has low screening ability to rule out sarcopenia. Clinically, this implies that individuals who test negative using the Arabic SARC-F need further investigation. Our sensitivity finding is lower than the sensitivity results reported by several studies [15, 24–26]. This could be because sensitivity is affected by the proportion of individuals who test positive for SARC-F and they were only 23.3% in our study. Another explanation of the low sensitivity of SARC-F is likely to be since most of the SARC-F items are on muscle function rather than muscle mass. However, our finding is similar to French, Spanish, and Brazilian SARC-F versions where sensitivity values were 36.0%, 35.6%, and 33.3%, respectively, based on EWGSOP definition [27–29]. Conversely, our sensitivity result was higher than the sensitivity values of the original English SARC-F (female = 3.8% and male = 9.9%), Turkish SARC-F (25.0%), and Korean SARC-F (25.3%) based on EWGSOP definition [30–32].

In the current study, the Arabic SARC-F had very good specificity (78.7%) based on the EWGSOP2 criteria. The high specificity (78.7%) makes the Arabic SARC-F appropriate for detecting older people who need further testing to confirm sarcopenia. This specificity result is lower than findings reported by Woo, et al. in the original English version (98.7% in males and 94.4% in females) [30]. However, the specificity result in our study is higher than those in German SARC-F (47.0%), Danish SARC-F (53.2%), and Vietnamese SARC-F (68.2%) [15, 26, 33], whereas Bahat et al. found that the specificity of Korean SARC-F was 81% which is close to our finding [31]. The discrepancies in results may mean that the SARC-F questionnaire has acceptable but varying validity and reliability when utilized with older persons in a variety of nations and languages.

Based on the EWGOSP2 criteria, our PPV and NPV results were 20% and 89.3%, respectively. These results are in concordance with previous validation studies which reported that SARC-F has low PPV but high NPV. Similar values were reported by Gade et al. with PPV (24.1%) and NPV (80.6%) [33]. Moreover, Gasparik et al. reported higher PPV (40.6%) but relatively similar NPV (85.4%) [25]. Our findings imply that the Arabic SARC-F has low screening probability (20%) to accurately identify people with sarcopenia. However, it has higher screening probability (89.3%) in identifying people who do not have sarcopenia. Our results are in line with EWGSOP2 findings [13] and with the meta-analysis findings which was conducted to investigate the screening capability of SARC-F in older people [34].

The low sensitivity of the Arabic SARC-F indicates that it may miss some individuals who have sarcopenia, which is a critical limitation. Clinically, this means that a negative result on the SARC-F should not be solely relied upon to rule out sarcopenia. Healthcare providers should consider additional screening and diagnostic tools, such as DXA, especially for patients who exhibit symptoms or risk factors of sarcopenia but test negative on the SARC-F. This approach ensures a more comprehensive assessment and reduces the risk of underdiagnosing sarcopenia.

On the other hand, the high specificity of the Arabic SARC-F makes it particularly useful for identifying individuals who are likely to have sarcopenia. In a clinical setting, a positive SARC-F result can prompt further diagnostic evaluation, such as muscle mass measurements or functional performance tests, to confirm the diagnosis. This specificity supports the use of the Arabic SARC-F as an initial screening tool in primary care or community settings, where it can help to identify individuals who need more in-depth sarcopenia assessments efficiently.

### 4.3. Health-Related Correlations

The Arabic SARC-F was also inversely correlated with health-related measures including SPPB, gait speed, grip strength, and positively correlated with chair stands test. The significant correlation between the Arabic SARC-F and SPPB, gait speed, grip strength, and chair stands can be explained by the negative impact of the decline in muscle mass/strength on function and physical performance. This finding is similar to other SARC-F validation studies' results [15, 25–29, 32, 33, 35–37]. This finding also indicates that there is already significant association between physical performance metrics and the Arabic SARC-F version.

We also found significant correlations between the Arabic SARC-F and BMI, SF-12, and MFIS. Physical, mental, and total QOL scores using SF-12 were all significantly correlated with the Arabic SARC-F. However, the highest correlation was reported between the SARC-F and the physical subscale of SF-12, an expected finding since the SARC-F questions focus on muscle function and physical performance. Another study examined the association between SARC-F and QOL using the control, autonomy, self-realization, pleasure scale (CASP-12) reported a significant association between SARC-F and QOL [38]. Furthermore, Parra et al. reported a significant correlation between SARC-F and QOL using the visual analog scale from the EuroQol (EQ-VAS) [28]. Moreover, another study found significant correlation between SARC-F and QOL using EuroQol-5 (EQ-5) [32]. However, what distinguishes this study from other similar studies is that it is the first study that investigated the association between SARC-F and SF-12 since it is shorter than other longer versions of QOL measures and more appropriate for use among the older adults.

The positive correlation between the Arabic SARC-F and the chair stands test, along with the significant associations with SF-12 scores, particularly the physical subscale, underscores the impact of sarcopenia on the QOL. Patients with higher SARC-F scores may experience lower physical functioning and overall well-being. This relationship is clinically relevant, as it suggests that improving muscle strength and physical performance could potentially enhance QOL, making the SARC-F a useful tool for monitoring patient outcomes over time.

The association between SARC-F and fatigue with its subscales including physical, cognitive, psychosocial, and total fatigue scores was also established in this study with the highest correlation between SARC-F and MFIS-physical. Our results were in line with a recent study by Lin et al. who examined the correlation between SARC-F and Pittsburgh Fatigability Scale (PFS) and found a significant correlation between SARC-F and physical and mental PFS, and the highest correlation was with the PFS-physical subscale. Moreover, Justine and colleagues who investigated the correlation between fatigue and sarcopenia measurements in older people in Malaysia found that SARC-F total score is correlated with fatigue severity using Fatigue Severity Scale (FSS) [39]. Based on our results, the significant correlations found between the Arabic SARC-F and fatigue levels indicate that clinicians should be attentive to the symptoms of fatigue in patients who test positive for sarcopenia. This awareness is crucial because fatigue can significantly impair QOL and daily functioning, and addressing it as part of the sarcopenia management plan could lead to better patient outcomes.

In the current study, no significant correlation was found between SARC-F and MOCA. However, this finding contradicts a previous study finding that reported a significant association between SARC-F and cognitive impairment using Mini-Mental State Exam (MMSE) [28]. Kim et al. also reported that SARC-F significantly correlated with cognitive impairment using MMSE [32]. The difference between our finding and the results reported by other studies might be related to the nature of cognitive instruments used in different studies as well as the sample characteristics differences. Although MOCA is a good screening tool for cognitive impairment, it has various results with different individual's characteristics and cultures [40]. In this study, participants' median age was 65 years which is lower than the age in other studies that found a significant correlation between SARC-F and cognitive impairments. Also, the median MOCA score in this study was 26 which indicates that most of participants had good cognitive functions and did not suffer from mild cognitive impairments. Based on those two reasons, we did not find a significant association between the Arabic SARC-F and MOCA.

These relationships provide valuable context for interpreting the SARC-F scores in clinical practice. For example, a patient with a high SARC-F score might not only be at risk of having sarcopenia but also likely to experience significant physical and emotional challenges. This broader perspective can guide more holistic care planning, ensuring that interventions address not just sarcopenia but also its associated health impacts.

### 4.4. Limitations

There are some limitations in our research study. We used BIA to measure skeletal muscle mass rather than more precise but expensive and invasive techniques such as DXA, CT-scan, and MRI. These methods are considered the gold standards for evaluating muscle mass and body composition due to their higher accuracy and reliability. The limitations of BIA might have affected the accuracy of skeletal muscle mass measurements, potentially leading to an underestimation or overestimation of sarcopenia prevalence in our study population. Future research should consider utilizing more precise methods like DXA or MRI for muscle mass assessment to validate the findings of this study and to further refine the diagnostic criteria of sarcopenia. Additionally, incorporating a combination of methods could enhance the accuracy and reliability of sarcopenia diagnosis. We also noted that there are a higher number of female participants in the sample, which may have impacted the SARC-F criteria, because males are more likely to choose responses that emphasize their physical capability, whereas females are more likely to choose responses that emphasize their need for others' care and support. One more limitation is the percentage of individuals diagnosed with sarcopenia representing a small percentage of the entire group investigated and this might be skewed against those who do not have sarcopenia.

The findings of this study are based on a sample from Jordan, which might limit the generalizability of the results to other Arabic-speaking populations. Cultural, social, and economic differences across Arabic-speaking countries may influence the applicability and interpretation of the SARC-F tool. To enhance the generalizability of the Arabic SARC-F, future research should consider validating the tool in different Arabic-speaking countries. Cross-cultural validation studies can provide insights into the tool's applicability across diverse settings and help in developing region-specific adaptations if necessary. Additionally, the study's sample may not accurately reflect the larger older adult population at higher risk for sarcopenia, given its high MOCA scores and median age of 65 years. This limitation should be more clearly acknowledged, as it may affect the applicability of the findings to populations with higher sarcopenia risk.

The low sensitivity of the Arabic SARC-F may lead to underdiagnosis of sarcopenia in clinical settings, particularly among populations with mild symptoms or early stage of sarcopenia. Clinicians should be aware of this limitation and consider using additional diagnostic tools and methods to confirm the presence of sarcopenia. Clinicians may consider incorporating other assessment tools, such as physical performance tests or muscle strength measurements, in conjunction with the SARC-F to improve diagnostic accuracy. Combining multiple assessment methods can provide a more comprehensive evaluation of sarcopenia and guide appropriate interventions.

## 5. Conclusions

The development of the Arabic version of the SARC-F questionnaire marks a significant advancement in the assessment of sarcopenia among Arabic-speaking populations, with the study demonstrating its excellent test–retest reliability and high internal consistency. Despite its low sensitivity in ruling out sarcopenia, the tool's high specificity makes it effective in identifying patients who may have sarcopenia. The translation and cultural adaptation, including the use of kilograms instead of pounds, were conducted according to the WHO guidelines, ensuring linguistic and cultural appropriateness. The high specificity of the Arabic SARC-F makes it a valuable tool in clinical settings, but its low sensitivity suggests the need for additional diagnostic measures.

Future research should focus on larger and more diverse sample sizes, using more precise diagnostic tools such as DXA, CT, and MRI, and validating the tool across different Arabic-speaking countries to enhance its cultural adaptability and generalizability. Additionally, prevalence studies of sarcopenia in Jordan and other Arabic-speaking regions are necessary to understand the condition's extent and inform targeted public health interventions.

## Figures and Tables

**Table 1:** Characteristics of participants (*N* = 86).

Variable	All participants
Age, median (IQR)	65 (8)
Gender, *N* (%)	
Male	35 (40.7%)
Female	51 (59.3%)
Education-level, *N* (%)	
High school or below	31 (36%)
Diploma	19 (22.1%)
University degree	36 (41.9%)
Smoking, *N* (%)	
Not smoking	76 (88.4%)
Smoking	10 (11.6%)
Fall last year, *N* (%)	
Yes	17 (19.8%)
No	69 (80.2%)
SARC-F (using cut-off score)	
No sarcopenia, *n* (%)	66 (76.7%)
Sarcopenia, *n* (%)	20 (23.3%)
BMI, mean (SD)	31.09 (0.05)
Number of cigarettes, median (IQR)	5.50 (12)
Number of diseases, median (IQR)	2.00 (3)
Number of falls, median (IQR)	1.00 (0)
Number of medications, median (IQR)	3.00 (2)
SARC-F question 1, median (IQR)	0.00 (1)
SARC-F question 2, median (IQR)	0.00 (0)
SARC-F question 3, median (IQR)	0.00 (0)
SARC-F question 4, median (IQR)	0.00 (1)
SARC-F question 5, median (IQR)	0.00 (0)
SARC-F total, median (IQR)	1.00 (3)
MFIS-physical, median (IQR)	8.00 (14)
MFIS-cognitive, median (IQR)	3.50 (12)
MFIS-psychosocial, median (IQR)	2.00 (3)
MFIS-total, median (IQR)	14.50 (25)
SF-12-physical, median (IQR)	64.29 (42.86)
SF-12-mental, median (IQR)	71.43 (33.33)
SF-12-total, median (IQR)	68.57 (31.43)
MOCA, median (IQR)	26.00 (4)
SPPB, median (IQR)	10.00 (2)
Speed (m/s), mean (SD)	1.08 (0.45)
Grip strength (right hand), median (IQR)	25.0 (1.22)
Grip strength (left hand), median (IQR)	24.6 (1.23)
Chair stand, median (IQR)	15.26 (8.75)
ASM, median (IQR)	17.89 (7.98)
ASM/ht^2^, median (IQR)	7.17 (1.82)
ASM/ht^2^ (using cut-off score)	
Sarcopenia, *N* (%)	11 (12.8%)
No sarcopenia, *N* (%)	75 (87.2%)

**Table 2:** Test–retest reliability of SARC-F item and total scores (*N* = 78).

Item	Mean	Range	ICC	95% CI
1	0.827	0.821–0.833	0.902	0.847–0.938
2	0.340	0.333–0.346	0.869	0.794–0.916
3	0.314	0.295–0.333	0.850	0.764–0.904
4	0.750	0.731–0.769	0.894	0.834–0.933
5	0.353	0.346–0.359	0.877	0.807–0.921
Total	2.583	2.526–2.641	0.926	0.884–0.953

**Table 3:** Distribution of samples using the Arabic version of SARC-F questionnaire and the EWGSOP2 criteria.

SARC-F	EWGSOP2 (ASM/ht^2^)		Total
	Sarcopenia	No sarcopenia	
≥ 4	4	16	20
< 4	7	59	66
Total	11	75	86

**Table 4:** The association between SARC-F total score and other variables (*N* = 86).

Variable	Spearman correlation
Appendicular segmental mass/high^2^	−0.179
Appendicular segmental mass	−0.272^∗^
Gate speed (m/s)	−0.396^∗∗^
Grip strength (right side)	−0.271^∗^
Grip strength (left side)	−0.244^∗^
Chair stand (second)	0.351^∗∗^
SPPB	−0.410^∗∗^
Age	0.074
BMI	0.270^∗^

Abbreviations: BMI = body mass index, SPPB = Short Physical Performance Battery.

^∗^ *p* < 0.05.

^∗∗^ *p* < 0.001.

**Table 5:** The association between SARC-F total score and domains of MFIS, SF-12, and MOCA.

Variable	Spearman correlation
MFIS-physical	0.511^∗∗^
MFIS-cognitive	0.492^∗∗^
MFIS-psychosocial	0.389^∗∗^
MFIS-total	0.537^∗∗^
SF-12-physical	−0.679^∗∗^
SF-12-mental	−0.520^∗∗^
SF-12-total	−0.631^∗∗^
MOCA	−0.190

*Note:* SF-12: Medical Outcomes Study Short Form 12, MOCA: Montreal Cognitive Assessment.

Abbreviation: MFIS = Modified Fatigue Impact Scale.

^∗∗^ *p* < 0.001.

## Data Availability

The data used in this study are available from the corresponding author upon request.
